# Action selection: A race model for selected and non-selected actions distinguishes the contribution of premotor and prefrontal areas

**DOI:** 10.1016/j.neuroimage.2010.02.045

**Published:** 2010-06

**Authors:** J.B. Rowe, L. Hughes, I. Nimmo-Smith

**Affiliations:** a Cambridge University Department of Clinical Neurosciences, CB2 2QQ, UK; b MRC Cognition and Brain Sciences Unit, CB2 7EF, UK; c University of Cambridge Behavioural and Clinical Neurosciences Institute, CB2 3EB, UK

**Keywords:** Response selection, Prefrontal, Cingulate, Race model, Decision making, fMRI

## Abstract

Race models have been used to explain perceptual, motor and oculomotor decisions. Here we developed a race model to explain how human subjects select actions when there are no overt rewards and no external cues to specify which action to make. Critically, we were able to estimate the cumulative activity of neuronal decision-units for selected *and* non-selected actions. We used functional magnetic resonance imaging (fMRI) to test for regional brain activity that correlated with the predictions of this race model. Activity in the pre-SMA, cingulate motor and premotor areas correlated with prospective selection between responses according to the race model. Activity in the lateral prefrontal cortex did not correlate with the race model, even though this area was active during action selection. This activity related to the degree to which individuals switched between alternative actions. Crucially, a follow-up experiment showed that it was not present on the first trial. Taken together, these results suggest that the lateral prefrontal cortex is not the source for the generation of action. It is more likely that it is involved in switching to alternatives or monitoring previous actions. Thus, our experiment shows the power of the race model in distinguishing the contribution of different areas in the selection of action.

Race models can account for many behavioural, perceptual and oculomotor decisions (Carpenter and McDonald, 2006; Gold and Shadlen, 2007; Ratcliff and Rouder, 1998) based on trial to trial variations in a race between alternative responses. In race models, and related drift diffusion models, activity of neuronal decision-units rises from baseline to a threshold that represents commitment to a response (Churchland et al., 2008; Roitman and Shadlen, 2002; Yang and Shadlen, 2007). Here we apply a race model to action selection when there were no overt rewards and no external stimulus to specify ‘correct’ actions. There are two lines of neurophysiological evidence to suggest that race models are relevant to decisions of this sort. Firstly, if monkeys are taught to base their decision on the direction of coherently moving dots, accumulating neuronal activity is found that reflects the decision even when there is no coherent motion and both choices are equally rewarded (Churchland et al., 2008; Roitman and Shadlen, 2002.). Secondly, the decision threshold for a selected action is constant, whether or not it is specifically cued.

Here we developed a race model and used fMRI to identify brain activations that reflect parameters of this model (see Fig. 1 and Methods). In our model, action selection emerges from the race between competitive decision-units, each associated with distinct action schemas for permitted responses (Norman and Shallice, 1980). Motor decision-unit activity rises from baseline to response threshold with a Gaussian rate distribution. Based on the parameters of these decision units, we estimated accumulated metabolic activity (EAA) for each trial. The trial-by-trial estimates of EAA informed the analysis of fMRI data. Critically, we were able to estimate the total demands of both the ‘winner’ and ‘losers’ of the race, using the Inverse Mills Ratio for truncated Gaussian distributions (losers are truncated by the ‘winner’ reaching the response threshold).

We chose a task that has frequently been used in the human imaging literature, with reproducible data on the brain regions that are involved. Human participants chose between manual responses where there were no overt rewards and there was no external stimulus to guide one action rather than another. This is associated with reproducible differential activation of dorsal prefrontal cortex, the pre-SMA and the intra-parietal cortex (Deiber et al., 1991; Forstmann et al., 2008; Frith et al., 1991; Rowe et al., 2005, 2008). However, the different roles of these regions are less well understood and activations may arise because of different cognitive operations even within such an apparently simple task (Lau et al, 2004).

Therefore, our fMRI analysis also included a categorical term that distinguished specified from selected responses, in addition to the EAA that is related to trial to trial variation in response decisions. This approach allows us to distinguish the selection between action schemas from other processes which, although temporally associated with selection, are not the mechanism of selection itself e.g. memory or monitoring of recent responses to modulate future response selection. Because these occur over a series of trials, we also performed a second study in which we measured activity for action selection on the first trial alone. Activity on this trial must reflect selection rather than memory.

The present study had three aims. The first aim was to test the application of the race model to the selection of human manual responses when multiple responses are permitted. The second aim was to see whether it can distinguish between the contributions of different cortical areas to response selection. The final aim was to understand how selection on one trial is influenced by the context of recent actions.

## Methods

### Subjects and task

For the first study, twenty healthy adults participated (age range 19–40, mean 26 years, 10 men). For the second study, fifty seven subjects participated (range 18–75, mean 43 years, 31 males). None had a history of significant neurological or psychiatric illness. The studies were given a favourable opinion by the local Research Ethics Committee and participants gave written informed consent.

**Fig. 1:**
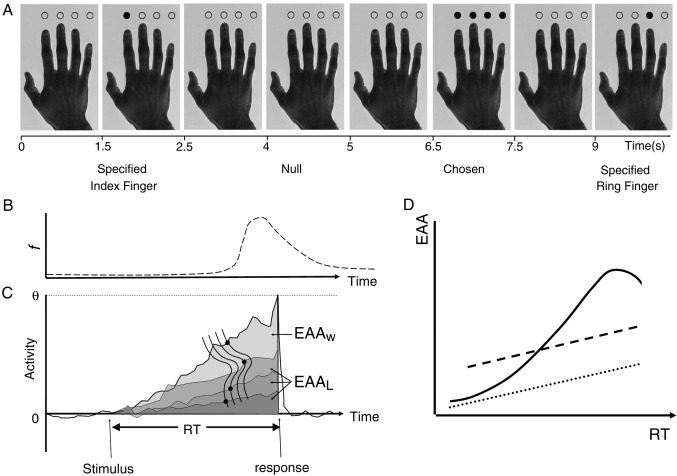
(A) The task required subjects to perform a specified button press (Specified condition) as indicated by a single dot above the corresponding finger of a picture of a hand, or to choose one of the four the possible button responses (Chosen condition). (B, C) schematic representations of the skewed distribution of reaction times (RT), arising from the Gaussian distribution of speed of response. A response results from a decision processor drifting between baseline activity 0 and a threshold *θ*. The estimated accumulated activity (EAA, grey shading) above baseline in the race model is a function of the threshold and RT and can be estimated for winners (EAA_w_) and losers (EAA_L_) in the race (see Methods). With four decision units drifting to threshold with a common underlying Gaussian distribution, the winner is the one at the faster (left) hand end of the rate distribution (upper black dot) whereas the other three losing decision units on this trial are at slower points on the rate distribution (lower three black dots). (D) Schematic representation of total EAA in relation to RT. The EAA for specified responses is linearly associated with the RT in the race model (dotted line). For chosen responses, the EAA is a non-linear function of RT (solid line), approximating the value for specified trials at very short and very long RTs, but greater than specified trials for intermediate RTs. The precise shape of this non-linear relationship depends on the subject-specific values of mean 1/RT and its variance. The effect of a categorical difference between trial types, due to an additional non-race ‘choice’ process, is illustrated by dashed lines.

The task is at first glance simple: subjects made button presses with one of four fingers of the right hand. In the main study, for a third of trials, a response was ‘specified’ in time and position by a filled in dot above the picture of a hand for 1 s (Specified condition). On another third of trials, the subjects chose which finger to press, in response to a similar visual cue in which all circles were filled in for 1 s (Chosen condition). See Fig. 1 for example cues. A third of trials were ‘null’ trials which included continuous presentation of the hand picture with no change in colour of the dots. The trial order was randomised, and stimulus onset asynchrony was 2.5 s. For the subsidiary study, the stimuli were similar, and the first response was either chosen or specified as above. Subsequent trials however differed for some subjects in terms of the trial order, being either blocked or randomised.

Despite the apparent simplicity of the task, we were specific in the supplementary instructions during training and immediately prior to scanning. For the Chosen condition, subjects were asked to “make a fresh choice on each trial using any of the four buttons, regardless of what you have done before”. We did not ask participants to make a random sequence, since this can have the paradoxical effect of increasing monitoring and constraining successive responses. We did not encourage or discourage any particular pattern, sequence or repetitions: these types of short structured sequences may occur by chance in a random sequence but are likely to be inhibited by subjects asked to ‘try to be random’.

### Behavioural data analysis

If responses were chosen at random with equipoise over four options, then the distribution of successive pairs would be even. The deviation from randomness can be assessed by Shannon's equitability index, the ratio of observed information per sequential response pair over the theoretical maximum observed information. For four responses, distributed over n sequential response pairs (*n* + 1 trials) this is given by:

$$E=H/H_{\text{max}}=\frac{\sum_{j=1}^{4}\sum_{i=1}^{4}\left(n_{\mathrm{ij}}/n\right)\ln\left(n_{\mathrm{ij}}/n\right)\;}{2\sum_{i=1}^{4}\left(n_{i}/n+1\right)\ln\left(n_{i}/n+1\right)}$$

where *n_ij_* is the number of trials in which response *j* followed response *i*, and *n_i_* is the number of trials with response *i*. With a finite number of trials, the Shannon index for sequential randomly generated specified responses is less than one, but it is even lower for chosen responses (see Results). Typically, repetitions of the same action are suppressed and switches to remote fingers on sequential choices are excessive. However, this suppression of repetition is less with our task than when under the instruction to make a random sequence (cf. Baddeley et al., 1998). Thus, free selection of action should not be equated with random behaviour. The subjects' Shannon equitability indices for chosen responses were used in a second-level parametric analysis of the differential BOLD response to chosen and specified trials.

The reaction times were expressed as the reciprocal RT (speed of response, 1/RT) because of their close approximation to Gaussian distributions (cf. oculomotor responses, Carpenter, 2004; Reddi et al., 2003). From the Gaussian distribution of the reciprocal RT, we determined the mean (= median) and the variance for each finger, under each condition, for each subject. These were then used as dependant measures in repeated measures ANOVA with response (4 levels, one for each button) and condition (Selection vs. Specified) as within-subjects factors.

### Estimation of expected accumulated activity (EAA) prior to response

The mean and variance of reciprocal RT were not significantly different between fingers. We defined each finger/button press to be governed by similar and independent decision processes (cf. Hanes and Carpenter, 1999) for which the drift rate of the decision processor is the main determinant of RT. This parallels previous analysis of oculomotor responses (Bruce and Goldberg, 1985; Carpenter, 2004; Reddi et al., 2003). Moreover, we include similar prior probabilities of each response, supported empirically by the distributions of responses across subjects with similar means and variances of reciprocal latencies for different responses within each condition. The independent decision processors may in principle be localised or distributed in the brain. We do not specify in advance whether they operate as single neurons, analogous to motion perception neurons (Roitman and Shadlen, 2002) or as distributed neuronal assemblies. We do specify that they are in competition, and that the race ends when one of the units reaches threshold with a stable linear upper bound (see Fig. 1).

There are therefore two steps to determine the expected neuronal activation from any trial, from which to inform the model of induced BOLD responses. The first step is to determine the parameters defining each decision-unit from the observed distributions of RT. For each of *k* decision processors (*D*_*d*_, *d* = 1,…*k*), accumulating activity to threshold *θ*, at a rate *β*_*d*_ drawn from a population of mean *μ*_*d*_ and variance *σ*_*d*_^*2*^, the winner *d*^⁎^ is the one for which *β*_*d*_, is maximum, with a winning rate *β*^⁎^ *= β*_d*_ and corresponding winning RT^⁎^ = θ/*β*^⁎^. For each of the *k* − 1 losers D_L_, *β*_*L*_ has a truncated normal distribution *β*_*L*_ ∼ N(*μ*_*d*_, *σ*_*L*_^*2*^ | *β*_*L*_ < *β*^⁎^). Formulae for the mean and variance of a truncated normal distribution are available. These can be compactly expressed in terms of the Inverse Mills Ratio (Mills, 1955) (IMR). In particular the expected value of *β*_*L*_ is

$$E\left[\;\beta_{\text{L}}|\beta_{\text{L}}<\beta *\right]=\mu -\sigma \left[\frac{\phi \left(\frac{\beta *-\mu }{\sigma }\right)}{\Phi \left(\frac{\beta *-\mu }{\sigma }\right)}\right]=\mu -\sigma IMR\left(z*\right)$$

where

$$z*=\frac{\beta *-\mu }{\sigma }\;\text{and}\;IMR\left(z\right)=\frac{\phi \left(z\right)}{\Phi \left(z\right)}$$

The second, step is to estimate the expected summed activity in all decision processes prior to threshold being reached (including winners and losers in the race model). To estimate the expected accumulated activity (EAA) across all units on each trial, prior to threshold being reached by the winner at RT*, we estimated the trial specific expected sum of activity in one winner and three loser units prior to response time. For each trial therefore, we estimate the total EAA as the sum of the winners expected accumulated activity EAA_W_ and the three losers' accumulated activity EAA_L_. On specified trials we equate the winner with the specified response, with *k* = 1 and *β*_*L*_ = 0, EAA_L_ = 0. On choice trials at the winning RT* to unit threshold θ, the *k*− 1 losers will have EAA_L_ given by,

$$EAA_{\text{L}}=\beta_{\text{L}}\text{RT}{*}^{\text{2}}/\text{2}\text{.}$$

To help intuit the relationship between EAA and RT, imagine a four horse race. If the winner is extremely fast, the other three horses will, on average, only just have left the start gate. In this analogy, the EAA corresponds to the sum of the energy expended by all four competitors by the time the winner crosses the line, and would approximate the energy expended by the winner alone. In contrast, if the winner is averagely fast, then the other horses would be expected to have also run much of the course, and the EAA would be the sum of the winning horse (a linear function of RT) plus a large contribution from the three losing horses (a non-linear function of RT).

### MRI data acquisition and analysis

A Siemens Tim Trio 3-Tesla scanner was used to acquire 155 BOLD-sensitive T2*-weighted EPI images (TR = 2000 ms, TE = 30 ms, FA = 788, 32 slices, 3.0 mm thick, in-plane resolution 3* 3 mm, slice separation 0.75 mm, sequential descending order). The first six images were discarded to allow for steady state magnetisation. Subjects also underwent high resolution magnetization prepared rapid gradient echo scanning (MP-RAGE: TR = 2250 ms, TE = 2.99 ms, FA = 98, IT = 900 ms, 256 _ 256 _ 192 isotropic 1 mm voxels). Data preprocessing and analysis used SPM5 and SnPM5 (www.fil.ion.ucl.ac.uk/spm) in Matlab 7 environment (R14, Mathworks, CA). fMRI data were converted from DICOM to NIFTII format, spatially realigned to the first image, and sinc interpolated in time to the middle slice to correct acquisition delay. The mean fMRI volume and MP-RAGE were coregistered using mutual information, and the MP-RAGE segmented and normalized to the Montreal Neurological Institute T1 template in SPM by linear and non-linear deformations. The normalization parameters were applied to all spatio-temporally realigned functional images, the mean and structural images, prior to spatial smoothing of fMRI data with an isotropic Gaussian kernel full-width half-maximum 10 mm.

Three first level models were used. Model-1, for 20 young subjects performing the first experiment of event related intermixed specified and chosen responses, was a first level general linear model including one regressor representing stimulus presentation (TASK), and a second regressor representing the EAA, spanning both trial types. Condition specific differences in trial-by-trial values of EAA reflect the performance of the race model. It is also possible that differences between the task conditions exist, which are not part of the race model. Such differences might be due to effects other than the decision process itself, and in order to evaluate this possibility we also included in our model a third regressor that categorically contrasted the two conditions (CvS: a type used in most previous studies, above). Error trials were modelled separately (errors occurred on specified trials only, when a subject made a different response to the one indicated). The model also used a high-pass filter with a cutoff of 128 s, and AR(1) modeling of temporal autocorrelations. Contrast images for effects of interest were made for entry to second-level analyses. The second type of first level model, model-2, resembled model-1 but in addition to EAA, it included mean-corrected absolute RT. This model was used to address concerns about the additional information given by the EAA/race-model approach over and above a more traditional analysis of RT covariance.

A third type of first level, model-3, was used to study the effects of first right hand finger moves, in the second study of 57 subjects. The first moves were either specified or chosen, with stimuli like those of the main experiment. However, subsequent moves included intermixed and blocked event types. The model included a regressor identifying the first move; a second regressor identifying a later single chosen trial (2–4 min into the experiment); a third regressor identifying a single later specified trial (2–4 min into the experiment). A fourth regressor modelled all other moves, parametrically modulated by the distinction between chosen and specified moves. The model also used a high-pass filter with a cutoff of 128 s, and AR(1) modeling of temporal autocorrelations. Contrast images for effects of interest were made for entry to second-level analyses.

A second-level model of group effects (random effects) from model-1 included three contrast images from each subject for the effects of task, EAA and the categorical difference between specified and chosen trials. These were included in a second-level ANOVA, adjusted for non-sphericity with dependence between measures and unequal variance. Covariance components are estimated using a restricted maximum likelihood algorithm, assuming similar effects over all suprathreshold voxels, and adjusting the statistics and degrees of freedom during inference. SPM(t) contrasts were then generated for contrasts of interest including simple ([1 0 0 ]) and conjunction contrasts ([1 0 0] AND [0 1 0]). A second-level model of group effects (random effects) from model-2was structurally identical, but included contrast images from model-2 (i.e. the EAA effects that are orthogonal to other regressors, including RT).

Second-level models based on model-3 from the second experiment used a multiple regression (ANOVA) model over contrast images of interest, distinguishing subjects that had begun with a specified trial from those that had begun with a chosen trial. For all second-level models, SPM(t) maps were generated from linear contrasts for each effect of interest, thresholded at *p* < 0.05 (False discovery rate). Liberal exploratory thresholds (uncorrected) are also presented where false negative results at standard threshold would be of particular relevance.

To correlate the redundancy of information in chosen moves, contrast images from model-1 were subject to statistical non-parametric mapping (SnPM5), correlating the activation difference between chosen and specified trials against the subjects' value of Equitability. SnPM was preferable for this correlation over subjects because of the distribution of Equitabilities and because we expected a monotonic but not necessarily linear relationship between activation and equitability. Pseudo *t*-tests generated SnPM(t) maps thresholded at *p* < 0.05 corrected by randomisation and re-sampling over 5000 permutations.

## Results

### Behaviour

First experiment: Twenty subjects were asked to press a button with the fingers of their right hand, in response to a visual cue during fMRI scanning (Fig. 1). Which button was pressed could either be specified by a cue or be chosen by the subject. The reciprocals of reaction times for chosen and specified conditions were distributed normally for each subject (one-sample Kolmogorov–Smirnov tests *z*-values ranging 0.43 to 1.13, mean 0.73, with associated *p*-values ranging 0.15 to 0.99, mean 0.65). After analysis of the distributions of reciprocal RT (see Methods) we did not include a fixed delay between trial onset and the start of the variable decision period, or after the decision period and motor execution. Repeated measures ANOVA of the subjects mean reciprocal RT for each finger (factor: finger, 4 levels) and each condition (factor: chosen vs. specified, 2 levels) confirmed that specified responses were slightly faster (mean rate selected 1.7 Hz, specified 1.9 Hz, SD 0.1 Hz, *F*_1,19_ = 43, *p* < 0.001). The difference in mean response latency corresponds to a difference in median RT of ∼ 60 ms. However, there was no difference between fingers (*F*_3,50_ = 1.9, ns, Greenhouse–Geisser correction) nor an interaction between different fingers and whether the response was specified or selected (*F*_3,48_ = 0.9, ns). The variance of the reciprocal RT was not different between fingers in the context of chosen responses (*F*_3,19_ = 2.5, ns, Greenhouse–Geisser correction) or specified responses (*F*_3,19_ = 2.1, ns, Greenhouse–Geisser correction).

As noted previously ‘freely’ chosen moves are often not random (Baddeley et al., 1998). Subjects in the first study did show redundancy (i.e. non-randomness or reduced observed information) of sequential chosen moves (mean 0.61 sd .0.14; see Fig. 3; compared with the maximal possible for infinite random sequence = 1.0, and the mean of synthetic random sequences of same length 0.84; *t*-test for difference between chosen and specified conditions *t* = 7.0, *p* < 0.001). We instructed subjects to “make a fresh choice on each trial”, and did not invite random responses (cf. (Baddeley et al., 1998) or (Jahanshahi et al., 2000) or impose other rules on how to choose e.g. we did not instruct subjects “to avoid the previous move” (Cunnington et al., 2006) or “avoid repetitive sequences” (Johns, 1996). Although some subjects may still have interpreted our instructions in terms of ‘randomness’, the behavioural evidence suggests this was less than previous studies. For example, whereas Baddeley et al. (1998) noted that repetitions of the same finger occurred only ∼ 10% as often as expected by chance, our subjects made repetitions at 93% of the chance rate. This was not significantly reduced in chosen vs. specified trials (chosen 93% vs. specified 96% of the response repetition rate expected within an infinite random sequence: paired *t*-test *t* = 0.19, df 19, ns).

### Functional magnetic resonance imaging

**Fig. 2:**
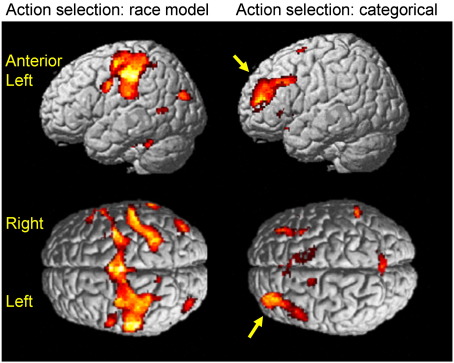
Activations associated with the predicted estimated accumulated activity under the race model (left) and a categorical distinction between choice and specified trials (right), from the first experiment. Activations (*p* < 0.05, FDR) are illustrated as rendered in a representative brain in standard anatomic space.

First experiment: We used a race model with four independent response decision processors (see Methods) to estimate the expected accumulated metabolic activity (EAA) associated with a response, whether that response was specified or chosen by each of the 20 subjects. This estimated activity supported an ‘informed’ model of the fMRI BOLD response. Thus, chosen responses emerge from the same essential competitive neuronal action schema as specified responses, without invoking a ‘selection module’. The activity of these action schemas reflect prospective action selection decisions, related non-linearly to RT. The EAA on each trial under the race model was correlated with activation in anterior cingulate cortex (ACC), supplementary motor area (SMA) and premotor cortex, and other regions shown in Fig. 2 and listed in Table 1, but not mid- or rostral-prefrontal cortex. These results were not substantially altered by the first level inclusion of reaction times in addition to EAA (Supplementary Fig. S1).

Our model also included a categorical parametric modulator of responses, defining the difference between the chosen and specified conditions. Such a categorical difference might represent the monitoring of responses before or after they are chosen, transient activations in working memory for prior moves, or switches from habitual to non-habitual responses. This categorical term was associated with greater activation of rostral and dorsolateral prefrontal cortex bilaterally (see Fig. 2 and Table 2). It is possible that a region may contribute to both types of processes — prospective action selection decisions and categorical task differences. We therefore performed a conjunction analysis of regions associated with both the decision-making model and a categorical difference. Only the ACC showed such joint activation (Table 3).

**Fig. 3:**
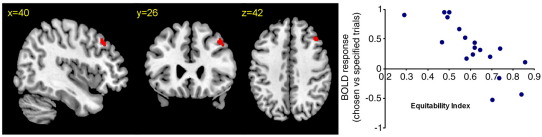
The categorical difference between chosen and specified trials (model-1) correlates with the redundancy of observed information for chosen trials (Shannon's index) in the dorsolateral prefrontal cortex (*p* < 0.05 corrected). Subjects with more activity here showed greater dependency on previous moves when ‘choosing’ an action.

If the categorical difference between conditions reflected the monitoring of the sequence of actions made, then this would permit a greater local contextual constraint on the selection process. Such temporally defined constraints are not explicit, but might nonetheless alter the deviations from the expected pattern of choices under random behaviours. We therefore correlated activity associated with chosen vs. specified responses against the subjects' mean observed information of chosen responses. A single region in right dorsolateral PFC (40, 26, 42, *t* = 5.36, FDR *p* < 0.05) correlated with the subjects' Equitability indices (see Fig. 3). No voxels showed the reverse correlation (*p* < 0.05 FDR or *p* < 0.001 unc).

**Fig. 4:**
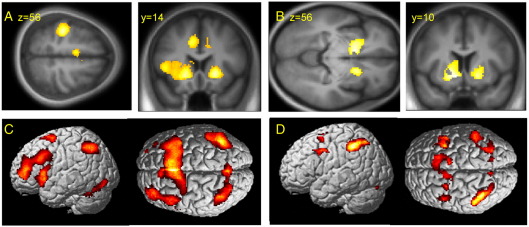
SPM(*t*) maps based on contrasts from the second experiment. (A) The very first move of the experiment is associated with activation of motor cortex and bilateral ventral striatum, left ventral premotor cortex and dorsal premotor cortex, insula, SMA and cingulate and cerebellum (*p* < 0.05 FDR). (B) Comparing first moves versus later moves (both specified and chosen) the ventral striatum is significantly more active bilaterally (*p* < 0.05, FDR). (C) The categorical contrast of chosen vs. specified trials was associated with activation of dorsal, polar and ventral prefrontal cortex (*p* < 0.05 FDR). (D) In none of these prefrontal regions associated with chosen vs. specified trials, was there a significant difference between subjects choosing their first move and subjects performing specified first moves even at *p* < 0.05 uncorrected.

Second experiment: If the categorical difference between chosen and specified responses (in lateral prefrontal cortex) was related to monitoring or switching, then this should not appear in the very first response, even if chosen. In a second analysis of a larger cohort of 57 subjects (see Methods) we therefore examined the very first response. The very first move of the experiment (both chosen and specified vs. baseline) was associated with activation of left motor cortex and bilateral ventral striatum and left ventral and dorsal premotor cortex, SMA and cingulate cortex, insula and cerebellum (*p* < 0.05 FDR, Fig. 4A). First moves in comparison with later moves were associated with greater activation of the ventral striatum bilaterally (*p* < 0.05, FDR, Fig. 4B).

The contrast of Chosen vs. Specified trials (all trials included) revealed activation of dorsal, polar and ventral prefrontal cortex (*p* < 0.05 FDR Fig. 4C). Even *single* chosen vs. *single* specified trials mid-way through the experiment were associated with activation of polar and ventral prefrontal cortex and parietal cortex (*p* < 0.05 FDR) reproducing previous studies that use a standard categorical contrast of Chosen vs. Specified responses, despite the potential low power of single trial regressors. However, in no prefrontal regions associated with chosen vs. specified trials, was there a significant difference between subjects choosing their first move and subjects performing a specified first move. For subjects choosing on their first move, there is no significant activation of mid or rostral prefrontal cortex even at *p* < 0.01 uncorrected (Fig. 4D). These results suggest that the lateral and polar prefrontal cortex does not play a significant role in the voluntary selection of first moves, despite their role in the selection of later moves.

## Discussion

Race models have effectively captured a variety of behavioural, perceptual and oculomotor decision-making processes (Carpenter and McDonald, 2006; Gold and Shadlen, 2007; Ratcliff and Rouder, 1998). For the analysis of fMRI, reaction times and other behavioural phenomena, race models have several advantages over simpler analysis of RT covariance or other methods to study action selection. They offer a parsimonious, generic and neurobiologically plausible mechanism by which actions can be selected on the basis of either a cued single action (specified) or a cued choice between actions. The models are strengthened by evidence of the neurophysiological properties of single neurons in perceptual and motor decision tasks. One can account for the distributions of RT with few parameters. In our case just two parameters were used to describe the Gaussian distributions of reciprocal RT across a group of subjects for this task while other tasks with complex cognitive and motivational factors can nonetheless be described efficiently by a small set of parameters. For action selection, the competitive race model also explains the absence of a longer RT when responses are selected rather than specified.

The analysis of our neuroimaging data based on the predictions of the race model revealed a clear distinction between the role of lateral prefrontal cortex and the roles of the premotor, motor, pre-SMA and cingulate cortex during action selection. The prefrontal cortex was associated with subject-specific bias towards switching to alternative sequential responses, and manifested within subjects as a categorical difference between chosen and specified responses. In contrast, the premotor, motor, pre-SMA and cingulate cortex were associated with prospective selection of responses, emerging from competition between action schemas. This distinction was confirmed by the analysis of selection of very first actions for which there was no monitoring or switching, and no activation of lateral prefrontal cortex. A similar distinction between lateral prefrontal cortex and the pre-SMA in voluntary action selection has been proposed previously (Lau et al., 2004). We suggest the race model as a candidate mechanism for such action selection, and extend this role to regions outside of the pre-SMA.

Importantly, our model included the same action schemas and same decision thresholds for both specified and chosen actions, without intercalating a modular selection process on choice trials. Despite the simplicity of the model, it explains key behavioural phenomena e.g. why the choice between actions adds little to reaction times (compared with a specified action) and may even result in shorter choice reaction times. It is also consistent with the earlier activations of pre-SMA for internally vs. externally generated moves (Cunnington et al., 2006). Moreover, the race model's identification of the pre-SMA as a component of prospective and competitive action selection is consistent with the observation that awareness of the chosen action may follow neurophysiological evidence of action selection, not precede it (Libet et al., 1983; Matsuhashi and Hallett, 2008) and does so in proportion to activity in pre-SMA and cingulate cortex (Lau et al., 2006).

We chose the action selection task because there are no explicit, implied or prospective differences in rewards associated with one or other chosen actions. In other tasks, where such differential rewards are known to subjects (Glimcher and Rustichini, 2004; Seymour et al., 2007 ; Seymour et al., 2004; Yang and Shadlen, 2007) or are likely (Daw et al., 2006; Dickhaut et al., 2003; Lee et al., 2005), then neuroeconomic theory and reward predictions can explain human choices. Moreover, there is evidence for a role of cingulate cortex in reward-based action decisions (Croxson et al., 2009; Rudebeck et al., 2008; Walton et al., 2007). This may be relevant even on trials in which one chooses between actions of *a priori* equal outcome (Walton et al., 2004). In this case, the context of recent differential rewards (feedback) is important since one interpretation is that subjects continue to engage in the assessment of the consequences of each choice, even when the equality of outcome was known for the current trial (Walton et al., 2004).

In our task, there was no difference in reward between responses during instruction, training or execution. However, there are at least two ways in which rewards may have contributed to our task. The first is a value associated with exploratory moves (Daw et al., 2006) such as the single first move in our second experiment. This first move was indeed associated with greater activation of the ventral striatum, which correlates with the value of upcoming actions (Morris et al., 2006) (Fig. 4), but this is unable to explain selection-associated cortical activation on later trials. The second is a possible implicit reward based on a self generated rule to match or non-match a previous response. However, an implicit reward according to adherence to a switch rule during sequential responses would not account for the pattern of cortical activations on first trials (second experiment).

Nonetheless, a generalised race model could incorporate differential values for each action (e.g. as learned or speculative action–reward associations) through reward-based bias of the race. The bias can either alter the baseline activity or rate of drift of competing action schemas. Indeed, our distinction between specified and chosen responses is an extreme case of such differential reward, since in the specified condition only the one action is ‘correct’. In our model, this difference between the correct and other responses is modelled as a difference in drift rates from baseline to threshold. We conclude therefore that while the race model is compatible with reward-based accounts of action selection, differential rewards are not *necessary* to select responses within a race model. The race model provides a mechanism by which a response can be chosen even when there is no difference in value of the alternatives.

The race between action schemas did not account for activation of lateral prefrontal cortex in choice trials. It is possible that the observed lateral prefrontal cortical activity reflects greater attention to action (Rowe et al., 2002) or attention to the selection of action (Lau et al., 2004). However, differential attention would not explain the lack of lateral prefrontal activation for first chosen moves. Another possible role of the lateral prefrontal cortex is working memory or monitoring of sequential responses, providing a context for the next move. Monitoring has been associated with activation of dorsolateral prefrontal cortex (Petrides, 2000). Previous studies have considered the potential confounding effects of working memory on choice tasks (Taylor et al., 2008; Lau et al., 2004). The medial frontal cortex including pre-SMA, but not the lateral prefrontal cortex, was identified as a correlate of choice, without differential monitoring or memory (cf. Table 3 in this study). Although supportive of our findings, it should be noted that Taylor et al (2008) studied chosen attention, not action.

Monitoring of sequential responses enables more response switching and there is evidence of a critical role of lateral prefrontal cortex in task switching (Rogers et al., 1998, 2000; Rowe et al., 2007). On self-ordered tasks for example, one must repeatedly switch to a new response. Lesions or transient perturbations by transcranial magnetic stimulation to mid dorsolateral prefrontal cortex impair such self-ordered tasks (Petrides, 1995) even without the need to monitor responses (Hadland et al., 2001). Accordingly, our subjects with less activity in lateral prefrontal cortex showed more random sequences or responses (Fig. 3).

The role of prefrontal cortex in switching over successive trials depends on the inter-trial interval. Imaging studies of action selection using intervals of 2–5 s found greater activity in lateral prefrontal cortex (Deiber et al., 1991; Forstmann et al., 2008; Frith et al., 1991; Rowe et al., 2008; Wiese et al., 2005; Lau et al., 2004). One study did not report activity of lateral prefrontal cortex (Cunnington et al., 2002) but in that paradigm the long intertrial intervals (8 to 24 s) may critically reduce the monitoring of prior moves and the switch to alternatives. In this context it is relevant that unconscious determinants of ‘free’ decisions in lateral fronto-polar cortex and medial parietal cortex extend as far as 10 s before the conscious decision (Soon et al., 2008), thereby including the intertrial intervals of most studies of action selection but not Cunnington et al. (2002). Thus, the unconscious shaping of a forthcoming action selection (Soon et al., 2008) may in part be due to monitoring of prior moves and switching to an alternative.

Switching to new responses is not necessary for a unique single response, and we found no evidence of lateral prefrontal cortical activation with selection of the very first action in the study. Other studies have examined chosen ‘single responses’ and yet found activations of lateral prefrontal cortex (Desmond et al., 1998; Nathaniel-James and Frith, 2002) but a critical factor in these earlier studies was that single responses were evaluated against a task rule or a remembered response set: no such accuracy or evaluation was necessary in our task.

There remain limitations of our method. Although the EAA is a non-linear function of RT (dependent on the relative values of mean 1/RT and its variance) it was possible that much of the variance attributed to EAA was explicable in terms of a linear RT effect for some subjects. We therefore ran the separate analysis which included trial-by-trial measures of both EAA and RT as covariates. The principal findings of the first study were not altered by inclusion of the RT covariate (Supplementary Fig. S1) and we therefore focus on the simpler model results. However, we cannot rule out other cognitive processes making contributing to the variation in RT, independent of the decision units that in our model are the principal generators of the EAA.

We adopted a relatively simple race model. This was characterised by a linear upper bound; no delay between stimulus and race onset; and a stable interval between baseline and threshold. These were heuristic approximations, motivated in part by the limited number of trials per subject, and yet sufficient to test the hypotheses of the study. Whereas other drift diffusion models with two opposing bounds are readily applied to two-choice tasks (Carpenter and McDonald, 2006; Gold and Shadlen, 2007; Ratcliff and Rouder, 1998; Bogacz et al., 2006) our task included four response options. Therefore, like the LATER model (Carpenter and McDonald, 2006), we proposed diffusion to a single upper bound only (response threshold) for a each response, with the advantage that multiple decision units can coexist for model multiple responses. Unlike the LATER model for reflexic saccades however, our limited behavioural dataset did not require the model to parameterise two separate populations of decision units each contributing to a given response.

Other approaches to multiple decision processes can be taken. For example, a four-choice decision may be considered as a pair of two-choice decisions using joint drift-diffusion models (Churchland et al., 2008). Alternatively, the parameters for multiple independent accumulators in a race model may vary if the estimation of accumulated activity is for the winning perceptual units (Ho et al., 2009). The latter approach was also used to generate predictors for the analysis of fMRI, and the functional anatomy of multi-model decisions. Alternatively, a three-layer neural network model enabling Bayesian decoding of stimuli has been developed that accommodates multiple or even continuous decisions (Beck et al., 2008). These sophisticated models have been developed to study serial decision processes of perception and cognition; complex behavioural phenomena arising from multiple sources of noise; and interactions between perceptual or response units. Often, the perceptual decision is mapped directly to a response (manual or saccade). More work is required to compare different models (Bogacz et al., 2006) adjusting for model fit and complexity, for both perceptual and action decisions. Our choice of model was motivated by the action decision in our choice trials, when the action is not directly specified by the perceived stimulus. Moreover, for our paradigm and task duration, these more complex models were unnecessary to explain principal behavioural phenomena and might over-fit the data.

Our model assumed that the competitor decision-units were independent of each other within each trial. This is consistent with our behavioural data, available neurophysiological data (Ratcliff et al., 2007) and previous estimates of accumulated activity supporting fMRI models of decisions (Ho et al., 2009). This independence does not exclude top down modulations of the decision units. Inhibitory competition between decision units, analogous to those observed in the visual system (Desimone and Duncan, 1995), might reduce the activity of ‘losers’ in a race, although this reduction could be offset by the metabolic demands of inhibition.

A final consideration is that contrasts based on single trial regressors in our second study may be underpowered. There are two reasons why low power is unlikely to account fully for the lack of activation in prefrontal cortex on first moves. First, with similar power, the equivalent contrast of ‘chosen vs. specified’ single responses mid-way through the experiment did confirm lateral prefrontal cortical activation. Second, we looked for prefrontal cortical activations on first moves at the very liberal threshold of *p* < 0.01 uncorrected. We infer that there is detectable prefrontal cortical activity with single chosen moves within a sequence of moves, but not a single first move.

In conclusion, we suggest that the lateral prefrontal cortex does not itself prospectively select an action but is critical for responses based on information in working memory when a switch to a different response is to be made. This supports the conclusion of Diamond and Goldman-Rakic that the ability to switch from a habit based on prior moves is granted by the prefrontal cortex and confers “...the freedom to choose and control what one does” (Diamond and Goldman-Rakic, 1989). This contrasts with response selection as an emergent property of competing neuronal assemblies in cingulate, premotor cortex and pre-SMA. This competition could be subject to bias arising from action–reward associations or top-down influences on action selection, but it can also unfold in the absence of distinguishing stimuli or differences in reward.

## Figures and Tables

**Table 1:** Areas in which voxelwise activation was greater in chosen than specified responses (FDR *p* < 0.05) excluding regions with a trend towards correlation with the race model (mask threshold *p* < 0.05 unc), reporting peaks separated by 15 mm or more (likely Brodmann's areas in parentheses).

Region (Brodmann area)	t	*x*	*y*	*z*
Inferior frontal gyrus (45)	4.93	48	20	4
Fronto-polar cortex (10)	5.03	− 40	52	12
	3.23	24	46	28
Middle frontal gyrus (46)	4.97	− 34	44	14
	3.65	38	30	34
	3.57	38	16	50
	3.23	24	46	28
Medial prefrontal cortex	4.62	8	24	42
Anterior cingulate	4.54	2	32	24
Superior frontal gyrus	4.56	14	4	66
	3.59	− 16	6	64
Orbitofrontal/polar cortex	3.98	20	52	− 12
Angular gyrus	4.96	60	− 40	48
Insula	5.15	32	28	− 6
	4.89	− 30	16	− 12
Intra-parietal cortex	3.81	− 34	− 46	36
Precuneus	4.31	14	− 64	42
	3.84	− 10	− 66	38
Prestriate cortex	3.84	32	− 80	− 10
Midbrain (nigra)	3.59	− 4	− 14	− 14

**Table 2:** Areas in which voxelwise activity correlated with the predictions of the race model (FDR *p* < 0.05) excluding voxels with a trend towards a categorical distinction between chosen and specified trials (mask threshold *p* < 0.05 uncorrected), reporting peaks separated by 15 mm or more.

Region	t	x	y	z
Sup. Frontal sulcus (caudal)	4.72	30	− 2	48
Inf. Frontal gyrus (caudal)	3.79	60	14	30
Dorsal premotor cortex	3.86	− 30	− 8	48
	3.64	− 28	− 22	64
	3.20	28	− 16	62
Ventral premotor cortex	3.48	− 54	0	30
Motor cortex	3.72	− 40	− 28	48
	3.90	46	− 28	48
Anterior cingulate	3.63	− 4	4	34
Cingulate	4.27	− 6	0	50
	3.85	− 6	− 16	50
	3.86	10	− 8	48
	3.28	8	− 24	48
SMA	3.65	6	− 4	64
Sensory cortex	3.84	− 64	− 24	40
	3.98	34	− 42	48
	3.35	− 34	− 36	62
Lateral pons	4.70	10	− 26	− 24
	4.21	− 8	− 26	− 24
Superior temporal gyrus	3.91	68	− 42	18
Lingual gyrus	3.98	22	− 50	2
Prestriate cortex	3.87	22	− 60	16
Fusiform gyrus	3.98	− 38	− 44	− 28
Gyrus recti	3.80	4	20	− 16
Thalamus	3.67	14	− 20	6
	3.65	4	− 22	2
Middle Occipital gyrus	3.48	42	− 76	24
	3.34	− 38	− 84	22

**Table 3:** Areas in which voxelwise activity correlated with both the predictions of the race model (FDR *p* < 0.05) and a categorical distinction between chosen and specified trials (FDR *p* < 0.05).

Region(Brodmann area)	t	x	y	z
Paracingulate/pre-SMA	5.29	− 6	12	48
	4.47	10	12	44

## Appendix A Supplementary data

Supplementary Fig. S1 SPM{*t*} map of the activations associated with expected accumulated activity (EAA) from two models in experiment one, rendered on a representative brain in normal space with a lateral view (A, D), top view with frontal lobes towards the left (B, E) and axial glass brain views with frontal lobes toward the left (C, F). In the left hand panels (A, B, C) are shown the results from model-2, in which the first level models included trial-by-trial parametric modulation by both EAA and the RT, as well as a categorical contrast of chosen vs. specified trials. In the right hand panels (D, E, F) are shown the results of model-1, without the RT regressor. While there are subtle differences between the statistical images, the broad pattern is the same and importantly the EAA remains informative even in the presence of RT.

## Footnotes

Appendix A Supplementary data associated with this article can be found, in the online version, at doi:10.1016/j.neuroimage.2010.02.045.
