# Supplementary figures and images for: Action selection: A race model for selected and non-selected actions distinguishes the contribution of premotor and prefrontal areas
Source: Neuroimage. 2010 Jun;51(2):888–96. doi: 10.1016/j.neuroimage.2010.02.045 (PMC2877799; doi:10.1016/j.neuroimage.2010.02.045)

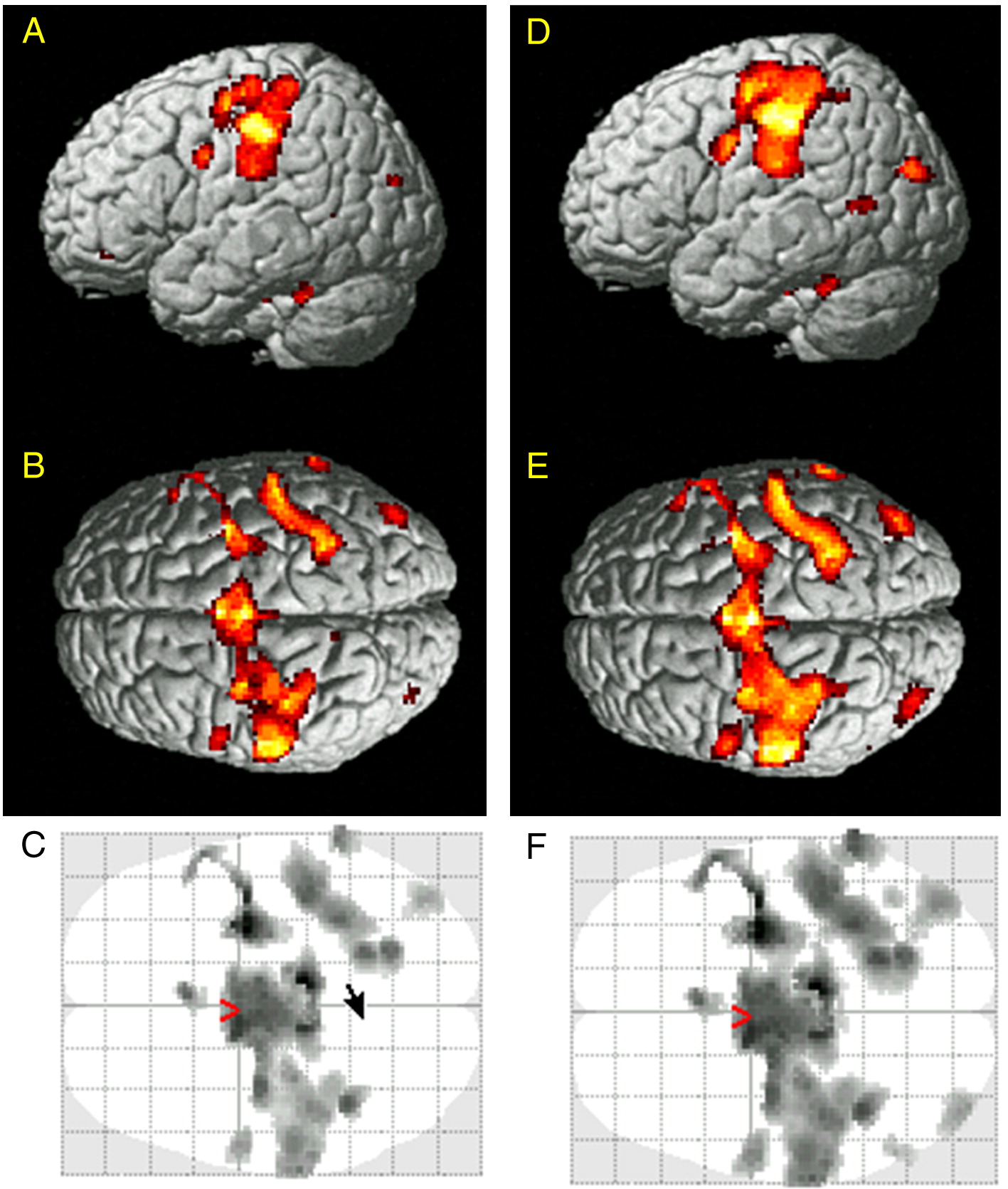

Supplement: Supplementary Fig. S1 — SPM{t} map of the activations associated with expected accumulated activity (EAA) from two models in experiment one, rendered on a representative brain in normal space with a lateral view (A, D), top view with frontal lobes towards the left (B, E) and axial glass brain views with frontal lobes toward the left (C, F). In the left hand panels (A, B, C) are shown the results from model-2, in which the first level models included trial-by-trial parametric modulation by both EAA and the RT, as well as a categorical contrast of chosen vs. specified trials. In the right hand panels (D, E, F) are shown the results of model-1, without the RT regressor. While there are subtle differences between the statistical images, the broad pattern is the same and importantly the EAA remains informative even in the presence of RT. [file gr5.jpg]
